# Experimental and Numerical Verification of the Railway Track Substructure with Innovative Thermal Insulation Materials

**DOI:** 10.3390/ma15010160

**Published:** 2021-12-26

**Authors:** Libor Izvolt, Peter Dobes, Marian Drusa, Marta Kadela, Michaela Holesova

**Affiliations:** 1 Department of Railway Engineering and Track Management, University of Žilina, Univerzitná 8215/1, 010 26 Zilina, Slovakia; libor.izvolt@uniza.sk (L.I.); peter.dobes@uniza.sk (P.D.); 2 Department of Geotechnics, University of Žilina, Univerzitná 8215/1, 010 26 Zilina, Slovakia; marian.drusa@uniza.sk; 3 Building Research Institute (ITB), Filtrowa 1, 00-611 Warsaw, Poland; 4 Department of Structural Mechanics and Applied Mathematics, University of Žilina, Univerzitná 8215/1, 010 26 Zilina, Slovakia; michaela.holesova@uniza.sk

**Keywords:** railway substructure, freezing of railway line construction, thermal insulation layer, composite foamed concrete layer, extruded polystyrene plate

## Abstract

The article aims to present the modified structural composition of the sub-ballast layers of the railway substructure, in which a part of the natural materials for the establishment of sub-ballast or protective layers of crushed aggregate is replaced by thermal insulation and reinforcing material (layer of composite foamed concrete and extruded polystyrene board). In this purpose, the experimental field test was constructed and the bearing capacity of the modified sub-ballast layers’ structure and temperature parameters were analyzed. A significant increase in the original static modulus of deformation on the surface of composite foamed concrete was obtained (3.5 times and 18 times for weaker and strengthen subsoil, respectively). Based on real temperature measurement, it was determined the high consistency of the results of numerical analyses and experimental test (0.002 m for the maximum freezing depth of the railway line layers and maximum ±0.5 °C for temperature in the railway track substructure–subsoil system). Based on results of numerical analyses, modified railway substructure with built-in thermal insulating extruded materials (foamed concrete and extruded polystyrene) were considered. A nomogram for the implementation of the design of thicknesses of individual structural layers of a modified railway sub-ballast layers dependent on climate load, and a mathematical model suitable for the design of thicknesses of structural sub-ballast layers of railway line were created.

## 1. Introduction

The growing urbanization of cities and agglomerations, which is associated with an increase in the number of people living an urban way of life, is simultaneously causing an increased demand for the transport of goods and people. Actually, support of transport must be conformed with the initiative of lower energy consumption, and lower noise production and greenhouse gas emissions, the so-called green transport [1]. Railways can therefore play an important role in meeting the needs of the population in travel for work, recreation, sports, or tourism, because rail transport is more ecological, economical, but it is also reliable and very safe for long distances. The year 2021 was declared as the “European Year of Rail”, with the aim of promoting the use of railway transport as one of the most sustainable modes of transport. In 2018, railway transport in the EU accounted for only 0.4% of total greenhouse gas emissions from transport; for comparison, road transport produced 71.8% [2].

Ensuring reliable and safe tracks for railways (especially for high-speed railways) requires components of the railway superstructure and substructure with sufficiently high resistance to deformation [3], which can be caused by traffic load (static and dynamic load [4,5,6]) and non-traffic load (climate load—water, frost, snow, etc. [7]). At present, as thermal insulation or reinforcement materials in sub-ballast or sub-base layers can be used various polymer materials e.g., expanded polystyrene (EPS) [8], polyurethane [9], extruded polystyrene (styrodur) [10,11,12], artificial aggregates, liapor with foam glass [13,14], and geosynthetic materials (geogrids, geo-mattresses) [15,16]. The expanded polystyrene (EPS) in contact with subsoil is also used as isolation under (shallow) foundation [17,18] and under road pavement foundation [19]. The installation of the XPS/EPS plates does not generate any chemical leaking and cause groundwater contamination, but these solutions are not ecological (polystyrene is difficult to recycle). Moreover, XPS/EPS products are very sensitive to the presence of organic solvents (but also to ozone and UV radiation), which can gradually, and eventually, degrade them. In an open system of railway construction, which can be driven by diesel locomotives, it is appropriate to keep this circumstance in mind. Therefore, in this study, foamed concrete panels was used as replacement of XPS/EPS plates. In addition, due to the fact that railway lines are linear structures, running through areas with various geological and hydrogeological conditions including weak subsoils, and increasing demands (higher speed, weight of transported cargo and transport reliability) requires to design trackbed substructures from a crushed aggregate of higher thickness. As the sources of natural aggregates are limited, in order to reduce the required thickness of the crushed aggregates sub-ballast, the article presents the possibility of applying a layer of composite foamed concrete (FC) and extruded polystyrene plates [20,21,22,23]. The layer of composite FC will be used to ensure sufficient bearing capacity and thermal resistance of the railway substructure, while the extruded polystyrene plates to ensure protection of the subgrade against freezing from track bench and from the slope side of the embankment. In the earlier research [7,11], authors determined that the application of extruded polystyrene railway sub-ballast layers increased the thermal resistance and had a negligible benefit for increasing the bearing capacity of sub-ballast layers. Thus, its combination with a layer of composite FC appears to be suitable and beneficial.

These materials have primarily one function in the construction of the railway line (thermal insulation or reinforcement), but the layer of composite FC can provide both functions simultaneously.

The development of new additives and the continuous modification of the composition of the foamed concrete (FC) mixture as well as easy pumping (even to great heights), seismic resistance, and coordinated deformation capacity [24,25] pushes this material forward and allows its wider and more efficient use. FC is widely used in the fields of thermal insulation [20], sound absorption [21,22], and fire resistance [23]. Moreover, foamed concrete has been used as non-structural elements in the construction of green buildings [26], a filler for bridge pillars to eliminate differential settlement [27], or in the underground construction of tunnels or subways [28,29]. In addition, there are studies on the production of prefabricated components [30], the design of building foundations [31], the design of airport buffer systems [32], but there are also numerical studies to examine the possibility of applying foamed concrete as a subbase layer for road structures [33,34,35]. Experimental studies of the possibility of application of reinforced foamed concrete mixtures on high-speed lines [36] or in transition areas for constructions of railway sub-ballast layers [37,38]. The application of composite foamed concrete mixtures in the body of the railway sub-ballast layers in the case of a low bearing capacity subgrade soil and at the same time due to its protection against frost has not yet been implemented or presented in foreign sources for applications in standard type railway transport.

The initiator of the research of the possibility of using the FC layer in the construction of the railway track substructure was the company iwtech Ltd. (London, UK) [39], which has been cooperating with the University of Žilina-Faculty of Civil Engineering (Žilina, Slovakia) for several years. Based on research cooperation agreement between them, thermal technical parameters [40], deformation characteristics [41,42], and dynamic characteristics [43,44] were determined for the foamed concrete layer and the possibility of its use in road subbase layers [45] or as a subbase of industrial floors [46] was assessed too. More than 10 years of scientific cooperation between them resulted in the granting of three patents in the field of application of composite foamed concrete in civil engineering structures with the aim of creating a subbase of high bearing capacity and with its minimized thickness and with uniform quality in the whole area and in the whole volume.

At present, research is focused on the possibility of applying a composite layer of FC in the sub-ballast layers of the railway substructure in terms of increasing its bearing capacity and thermal resistance. Railway temperatures are analyzed mainly in railway tracks [47,48,49], and there is only information about the subgrade temperatures [50]. Therefore, this study contains the results of experimental activities aimed at assessing the bearing capacity of the railway substructure with built-in composite layer of foamed concrete, comparison of freezing of railway line structure with modified composition of railway sub-ballast layers determined experimentally and numerically, realized proposal of modified structure of sub-ballast layers of railway substructure (through application of composite foamed concrete in combination with extruded polystyrene boards), and a method for designing structural thicknesses of a modified sub-ballast layers using a nomogram or a mathematical method.

## 2. Characteristics of Tested Materials and Used Methods

### 2.1. Foamed Concrete Development

In older sources, foamed concrete was often confused with aerated concrete or air-entrained concrete. However, Van Dijk’s definition of foamed concrete is a cement slurry in which at least 20% of foam is entrained into the plastic mortar (per volume) [51]. This definition clearly distinguishes the material from aerated concrete [52,53] or air-entrained concrete [54]. In reality, the FC should be called mortar, as no coarse aggregate is usually used for its production, and therefore, it is possible to achieve its very low bulk density (up to 75 kg/m^3^). Its density is usually regulated by partial or complete replacement of fine aggregate by means of foam [55].

Axel Errikson was the first company to patent Portland cement-based FC in 1923 [56]. However, detailed research concerning the production of foamed concrete, its composition and properties was not carried out until the 1950s and 1960s [56,57,58,59]. On the basis of this research, new admixtures and additives were developed in the late 1970s and early 1980s, which allowed the commercial use of foamed concrete in the construction industry, in particular for filling empty spaces and creating insulation [51]. Until the mid-1990s, FC was considered weak, poorly resistant and with high shrinkage properties, which was eliminated by the development of foaming agents based on synthetic enzymes, foam stability additives, and specialized foam forming and mixing equipment [57,58]. Moreover, the used components to production of foamed concrete mixture significantly affect its parameters [59,60,61]. Ma and Chen [61] used the magnesium phosphate cement to preparation of the foamed concrete. Falliano et al. [62] determined the higher compressive strength for foamed concretes based on protein foaming agent than synthetic. The opposite results were obtained by Sun et al. [63]. The effect of the synthetic foaming agent on the properties of hardening foamed concrete was presented in detail by Kadela et al. [64]. The use of a foaming agent is related to the air-void [63,65]. The effect distribution and size of the pores was presented, e.g., in [60,66,67]. Namsone et al. [68] determined that using expanded glass granule reduced the density of FC samples by 54% and 28-days compression strength (about 33%) compared to reference mixture. Zhang et al. [69] analyzed the effect of phenolic particles on mechanical and thermal conductivity and they achieved the best performance indexes for 20% the substitution of phenolic particles content, 5% foaming agent, 1% foam stabilizer, and w/c ratio equal 0.53. The used of waste materials as components of foamed concrete mixture (common as replacement aggregate or cement, and as fiber) was analyzed by other scientists. Dhir et al. [59], Chung Y-S. et al. [70], Kadela et al. [71], and Rommel et al. [72] used fly ash as a cement replacement, while Lesovik et al. [73] used a composition of Portland cement, opoka marl, and fly ash as a composite binder. Wongkvanklom et al. [74] investigated the impact of foam content on mechanical, thermal, and sound properties of foamed concrete made with 15 M NaOH solution, sodium silicate/NaOH ratio (NS/NaOH) of 1.00, sand/ash ratio of 1.25, liquid/fly ash ratio of 0.4. Similarly to ordinary concrete, there is a possibility of cracking [75] under the influence of the acting load (low tensile strength) and, as a result, the need to strengthen, e.g., by using fibers [76,77,78,79,80], CFRP [79], or mesh [80]. Moreover, in recent years, FC has been used extensively in various areas of construction around the world, such as in Germany, Great Britain, USA, Canada, and Brazil [81,82,83]. Due good mechanical and thermal properties, foamed concrete is used in various type of panel [70,84]. In addition, foamed concrete was used by Kadela et al. [85,86], Drusa et al. [87,88], Tian et al. [35], and Lee at al. [89] to transfer the load from the pavement or floor to the subsoil.

### 2.2. Properties of Used Materials

Table 1 presents the building materials used into the experimental field representing a modified structure of railway sub-ballast layers with built-in reinforcing and thermal insulation layer of composite foamed concrete. The designation FC 600 represents a modification of FC with a density of 600 kg/m^3^ (in the dry state). The basic physical and strength parameters of used materials are presented in Table 1. The methodology of this properties determination was the following:

(a) Density: The bulk density of aggregate was tested on three samples for each type of aggregate according to ASTM C29/C29M-07 [90]. The dry density of hardened samples of foamed concrete was measured on three samples with size of 150 mm × 150 mm × 150 mm according to EN 12390-7:2019/AC:2020 [91].
(b) Modulus of elasticity and Poisson’s ratio of foamed concrete were determined on the samples with dimension of 150 mm and height of 300 mm according to EN 12390-13:2021 [92]. Three samples were used.
(c) Compressive strength of foamed concrete was measured on samples 150 mm × 150 mm × 150 mm, according to EN 12390-3:2019 [93] using infraTest Compress Test Machine (Infratest Prüftechnik GmbH, Brackenheim, Germany) with force range 0–2000 kN. Three samples were used.
(d) Flexural strength of foamed concrete was tested on samples of 100 mm × 100 mm × 500 mm according to EN ISO 12390-5:2019 [94] using infraTest Compress Test machine with DigiMaxx C30 (FORM+TEST Seidner&Co. GmbH, Riedlingen, Germany) with infraTest double roller 35-0170. Three samples were used.

Table 1 presents an average values for each obtained properties.

The structural composition of the railway sub-ballast layers of the experimental field in question was designed according to Slovak railway standard [96], but a part of the sub-ballast layer composed of crushed aggregate was replaced by a composite layer of modified FC 600 specification of RW reinforced in its lower part with basalt reinforcing mesh ORLITECH^®^ Mesh (Orlibit Ltd. Osík, Litomyšle, Czech Republic) [97] with mesh size of 100 mm × 100 mm applied to Geofiltex 63/20 T geotextile (Mitop JSC, Mimoň, Czech Republic). The composition of foamed concrete FC 600N/RW (iwtech Ltd., Trenčín, Slovakia) for 1 cubic meter was:

- cement class CEM II/B-S 32.5 R—317 kg,
- sand fr. 0/2 mm—210 kg,
- water—160 kg,
- foam concentrate iwtech FC1 (iwtech Ltd., Trenčín, Slovakia)—1.56 kg.

**Figure 1:**
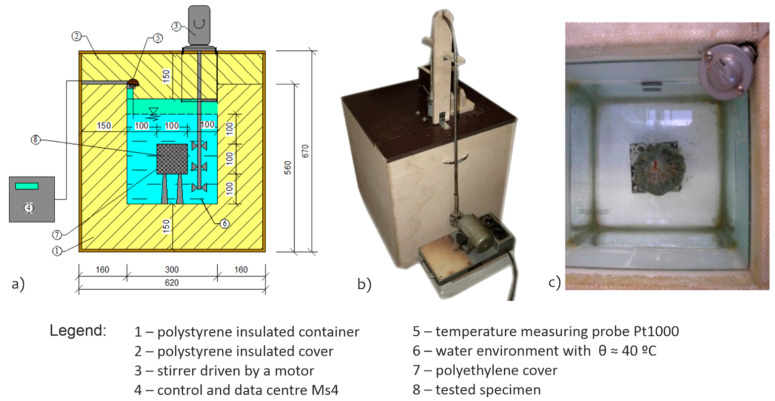
Calorimetry (**a**), determination of specific heat capacity of crushed aggregate (**b**), photo of the inside of the calorimetry (**c**).

**Figure 2:**
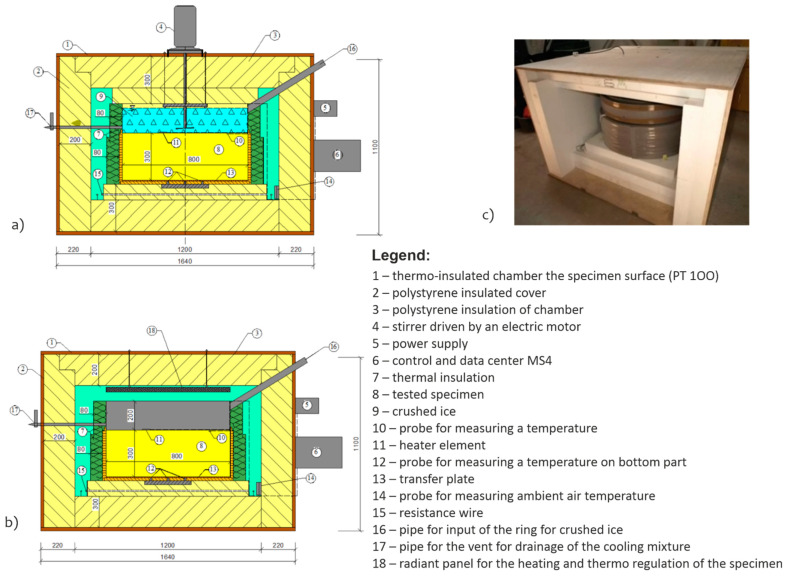
Device for recording the time interval of the specimen freezing, cross section of device in the mode of freezing specimen by crushed ice (**a**), cross section of device in the mode of heating (**b**), photo of device (**c**).

The thermal physical parameters (specific heat capacity and thermal conductivity coefficient) were determined in the laboratory using calorimetry of own construction (Figure 1a) and the method of determining the freezing time interval of the structural layer, see Table 2. The methodology of determination of specific heat capacity (Figure 1) and thermal conductivity coefficient (Figure 2) was described in detail in [98,99].

### 2.3. Methods of Testing in Experimental Field

#### 2.3.1. Description of Experimental Field

The possibility of applying a FC composite layer in the railway substructure is thanks to the increase of its bearing capacity and thermal resistance. An experimental field was built outside of the UNIZA campus at a scale of 1:1.

FC 600 class foamed concrete was manufactured according to the procedures and recommendations of iwtech Ltd. For the industrial production of FC, the technical foam generator series GFM8 (iwtech Ltd., Trenčín, Slovakia) at the concrete plant was used.

**Figure 3:**
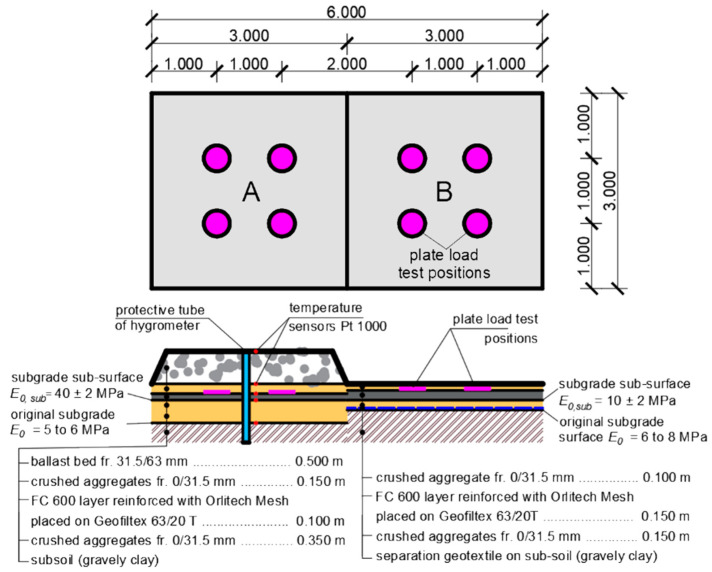
Experimental field—segment markers, material composition, localization of static load tests, and built-in temperature sensors.

The design of a modified structural composition of the sub-ballast layers is intended to increase the bearing capacity and thermal resistance of the structure, save natural materials (most often crushed aggregates), and also reduce the overall structural thickness of the sub-ballast layers. The structural and geometrical arrangement of the experimental field as well as the material composition of the individual structural layers of the modified sub-ballast layers are evident from Figure 3. The experimental field with its structural and geometric arrangement follows the experimental field characterized in [7,11], where extruded polystyrene with the trade mark Styrodur 2800C (BASF SE, Ludwigshafen, Germany) was applied in the structural composition of the sub-ballast layers. However, it has been shown that extruded polystyrene has a negligible effect on increasing the bearing capacity of the sub-ballast layers.

In terms of construction, the experimental field is divided into two segments (A, B), which are distinguished by different values of bearing capacity at the level of the subgrade sub-surface and the resulting different thickness values of the composite layers of foamed concrete. The subgrade sub-surface in the structure is the surface of the layer of crushed aggregate that was applied to the original subgrade surface. Segment A is characterized by the deformation-resistant subsoil, where conditions of satisfactory bearing capacity of the subgrade were expressed at the level of the subgrade sub-surface, expressed by the value of static modulus of deformation *E*_0,*sub*_ = 40 ± 2 MPa, while a 10 m thick composite layer of foamed concrete was embedded in the sub-ballast layers construction. Segment B is characterized by a low deformation-resistant subsoil, where at the level of the subgrade sub-surface conditions of unsatisfactory bearing capacity were created, expressed by the value of static deformation modulus *E*_0,*sub*_ = 10 ± 2 MPa, while a 0.15 m thick composite layer composed of foamed concrete was built into the structural composition of sub-ballast layers. The deformation modulus was determined by using Plate Bearing Test Equipment 100 kN (Fröwag GmbH., Obersulm-Eschenau, Germany) according to regulation TS4 [100] diameter of 300 mm. In order to protect the composite layer of foamed concrete from mechanical damage due to the pushing of the particles of ballast bed into its surface (due to the effects of dynamic effects from the passage of trains), a protective layer of crushed aggregate of fraction 0/31.5 mm was established on its surface. In segment A, in contrast to segment B, a ballast bed layer composed of gravel of fraction 31.5/63 mm is established on the sub-ballast layers. A built-in protective tube for the hygrometer (blue rectangle in Figure 3) together with Pt1000 temperature sensors (red circles in Figure 3) were also installed in Segment A, as this segment also serves to obtain input parameters for numerical modelling of the thermal regime of the substructure.

#### 2.3.2. Determination of Bearing Capacity of the Modified Railway Sub-Ballast Layers

**Figure 4:**
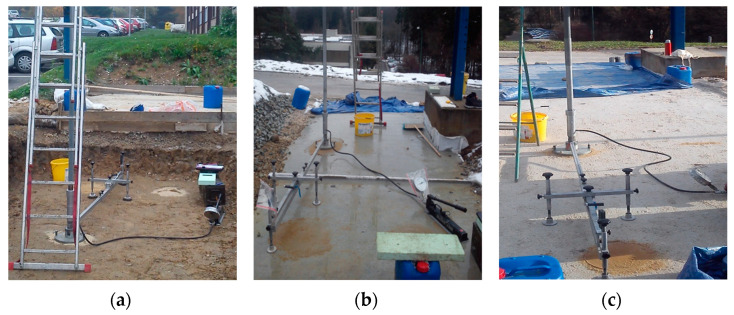
Experimental field—load capacity test on (**a**) the subsoil, (**b**) layer of foamed concrete, and (**c**) ballast bed.

The identification of the bearing capacity of the modified structure of the sub-ballast layers was realized on the experimental field by means of static load tests (Figure 4). These tests were performed according to the methodology specified in regulation TS4 [100] valid for the Slovak railways. They were carried out on the surface of each structural layer of the modified sub-ballast layers (subgrade, subgrade sub-surface, composite layer of FC and layers of crushed aggregate). As part of the static load test, a rigid circular load plate with a diameter of 300 mm was pushed successively in two load cycles into the surface of the above-mentioned structural layers with the value of 0.20 MPa being used as the maximum contact stress. Bearing capacity measurements were performed during the construction of the experimental field and subsequently in the second phase after two winter periods. The measured quantity was the static deformation modulus *E_mat_*, which was determined from the relation:

(1) $$E_{mat}=\frac{1.5\cdot p\cdot r}{y}\left[\mathrm{MPa}\right]$$

where:

*p*—is surface pressure acting on the plate [MPa],

*r*—is radius of the loaded plate [m],

*y*—is total average displacement of the loading plate found in the second cycle [m].

#### 2.3.3. Determination of Climate Characteristics of the Modified Structure of the Sub-Ballast Layers

Within the experimental field, specifically in segment A, the freezing of the railway structure (freezing depth *D_F_*) is monitored by means of five Pt1000 temperature sensors (Figure 1) located in the axis of segment A. Temperature sensors which are built into five different construction levels (surface of the ballast bed, sub-ballast upper surface, top and bottom surface of the composite layer of FC, surface of the original subgrade), record the instantaneous temperature with a periodicity of 30 min. Another temperature sensor is located 2.0 m above the surface of the surrounding terrain and is used to measure the air temperature, which is needed for the subsequent determination of the air freezing index *I_F_* and the average annual air temperature *θ_m_*. So far, four winter periods have been recorded in the experimental field and their determined thermal parameters (*θ_s_*_,min_—minimum mean daily air temperature during the winter period, *θ_m_*—average annual air temperature, *I_F_*—air freezing index, *I_Fs_*—surface air freezing index, *D_F_*—depth of freezing of the railway line structure) are given in Table 3.

**Figure 5:**
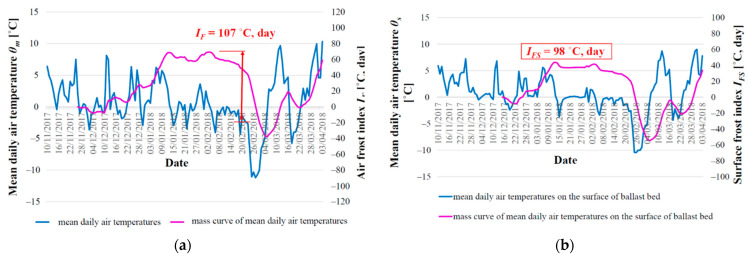
Graphical representation and method of evaluation of climate characteristics of the winter period 2017/2018 by air freezing index (**a**); surface air freezing index (**b**).

**Figure 6:**
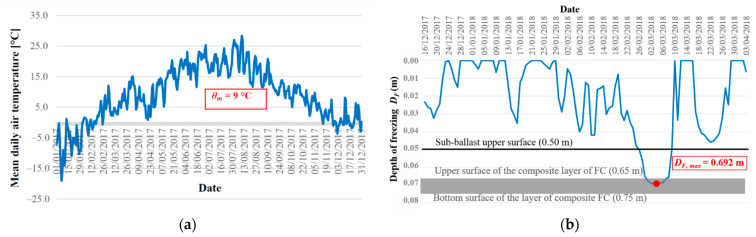
Graphical representation and method of evaluation of climate characteristics of the winter period 2017/2018—average annual air temperature *θ_m_* (**a**); freezing depth of the structure *D_F_* (**b**).

Figure 5 and Figure 6 depict the climate parameters for the winter period 2017/2018, which was the most unfavorable in terms of the achieved depth of freezing of the railway structure. This period will be used in this study in the comparison of the results of freezing depth of the railway structure obtained by means of experimental and numerical method.

#### 2.3.4. Numerical Thermal Analysis of Structures

**Figure 7:**
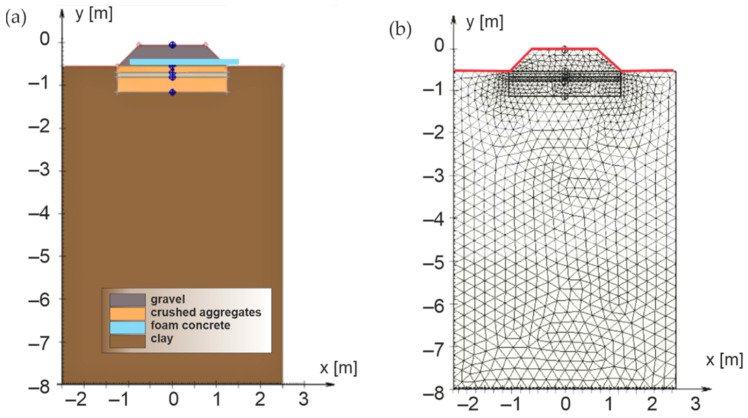
Numerical model of segment A of the experimental field (**a**) cross-section, (**b**) mesh.

The numerical thermal model of segment A of the experimental field (2D state—Figure 7) was created using the SoilVision software v. 2009, specifically using the *SVHeat* program v. 2.4.29 [101]. Within the numerical model, five areas representing individual material layers were defined (ballast bed, protective layer of crushed aggregate, composite layer of FC, levelling layer of crushed aggregate for the establishment of a subgrade and a clay subsoil) and their properties were specified. Input data of numerical modelling (material and thermo-physical properties) were obtained by monitoring in the experimental field, respectively by measurements in laboratory [102]. The material characteristics of the numerical modelling are evident from Table 2. The specific thermal conductivity is entered in the program for materials in the dry state and the coefficient of thermal conductivity depending on their humidity. The moisture content of the individual materials was defined on the basis of real values in the experimental field using the non-destructive TDR method (clay and crushed aggregate) or by the destructive method (sampling and drying) for materials where the TDR method (composite FC and ballast bed) could not be used. The moisture of the composite layer of foamed concrete was determined after two winter periods within the dismantling of segment B of the experimental field. The results of measurement are shown in Table 4. The temperature of materials of individual layers was entered as a real measured temperature in the experimental field on 31 December 2016 (Table 4), as the numerical modelling used the winter period 2017/2018 (reaching the highest freezing depth *D_F_* for the observed period—see Table 3) and the average annual air temperature *θ_m_* for year 2017 (coldest year). In addition to the parameters characterized in Table 4, it was necessary to define the maximum degree of saturation of individual materials and boundary for water freezing (freezing temperature, thawing temperature and method for determining the characteristic freezing curve of water) for individual material areas of the numerical model.

The boundary conditions for displacements are specified as standard fixities, under which full fixation (embedment) is set at the lower boundary of the overall geometry, and sliding supports at its vertical boundaries. On the area surface (red line in Figure 7b), the measured temperature was used as the boundary conditions. The temperature determined in segment A of the experimental field for 2017 (mean daily air temperatures characterizing the respective average annual air temperature *θ_m_* = 9 °C) and for the winter period 2017/2018 (mean daily air temperatures characterizing the respective air freezing index *I_F_* = 107 °C, day) were used in the numerical thermal modelling (see Table 3). The first day of the numerical model is marked as TIME = 1 day (real date 1 January 2017) and the last day is marked as TIME = 455 day (real date 31 March 2018), which is the 9th day after the last negative mean daily temperature in the winter period 2017/2018. The range of days from TIME = 1 day to TIME = 365 day characterizes the year 2017 in the numerical model and the entered environmental characteristics (mean daily temperatures) for these days are used to monitor the influence of the average annual air temperature on freezing of the railway structure. The range of days TIME = 331 day to TIME = 446 day characterizes in the numerical model the winter period 2017/2018 consisting of several frost periods (frost period is characterized by at least three consecutive days with negative mean daily air temperature) and entered environmental characteristics (mean daily air temperatures) for these days are used to monitor the effect of the air freezing index *I_F_* on freezing of the railway structure. Climate conditions (defined thermal characteristics) were assumed in the numerical model on the edges of material areas that are in contact with the air (edges of the surface of the ballast bed and the edges of the surrounding terrain). Within the climate condition, it is still necessary to define so-called *nf factor* (expresses the dependence between the air temperature and the temperature on the surface of the ballast bed) [101]. In this case, the value *nf* = 0.8 was used for this winter period, which is the value determined within the experimental monitoring. For the cases of winter periods when the snow cover was removed and outside winter periods, the value *nf* = 1.15 was used.

After entering the input parameters, it was possible to calculate the freezing of the structural layers of the railway line in the numerical model, which was realized using the FlexPDE v.2.4.29 program with the specified time step of the solution 0.1 per day. This program uses the infinite element method in solving complex differential equations and the ACUMESH program is used to visualize the results of the solution [103]. The grid generation module constructs a triangular (3, 6, or 9-node triangles) network of finite elements across any two-dimensional domain of the model. The network generator allows for spatially varying node densities to focus on areas of structural detail.

## 3. Results and Discussion

### 3.1. Bearing Capacity of the Sub-Ballast Layers

Figure 5 shows the average values of four measured values of the static deformation modulus *E_mat_* determined from the measured values on the surfaces of the individual structural layers of the modified structure of the sub-ballast layers. It should be noted that segments A and B differ from each other not only by the different bearing capacity determined at the level of subgrade sub-surface, but also by the different thickness of the composite layer of foamed concrete (0.10 and 0.15 m, respectively).

**Figure 8:**
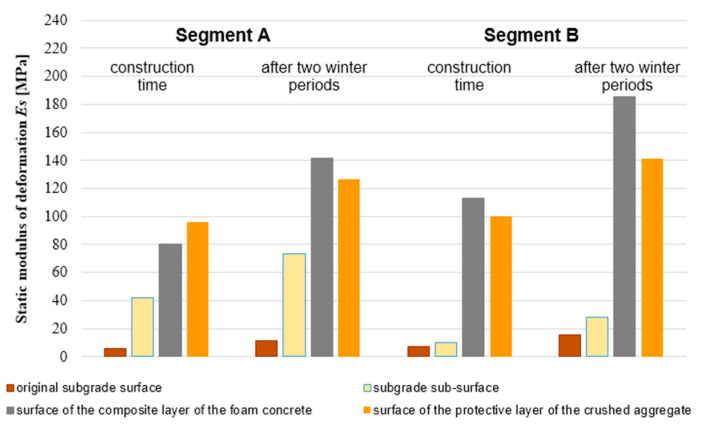
Average values of static deformation modulus *E_mat_* on the experimental field in segment A (with lower bearing capacity at the subgrade sub-surface and thickness of the composite layer of foamed concrete—0.10 m) and segment B (with higher bearing capacity at the subgrade sub-surface level and thickness 0.15 m of the composite layers of FC) during construction and after two winter periods.

From Figure 8, it is clear that in the case of deformation-resistant subgrade surface (static deformation modulus *E*_0_ = 40 ± 2 MPa), it is possible to double the value of static modulus of deformation measured after 28 days of its maturation by applying a composite layer of foamed concrete 0.10 m thick (*E_mat_* > 80 MPa). This value is above the required bearing capacity for the subgrade according to [96,100]. After 2 winter periods, up to 3.5 times the value of the original static modulus of deformation (*E_mat_* > 140 MPa) were identified on the surface of the composite foamed concrete. In the case of a low bearing capacity subgrade surface (static deformation modulus *E*_0_ = 10 ± 2 MPa), it is possible to increase the value of the static modulus of deformation measured after 28 days of its maturation and find values up to 10 times the original values by applying a composite layer of foamed concrete 0.15 m thick (*E_mat_* > 100 MPa). After two winter periods, in the case of this structure, an up to 18 times increase in the value of the static modulus of deformation (*E_mat_* > 180 MPa) was identified on the surface of the composite foamed concrete (FC). The obtained values confirm the legitimacy of using a foamed concrete layer to strengthen the substrate, and thus reduce subgrade settlement, which is particularly important in the case of high-speed railways [102,103,104,105,106]. Moreover, this solution can be used as an alternative to other applied solutions to strengthen the substrate [15,107,108].

### 3.2. Validation of Thermal Numerical Model

**Figure 9:**
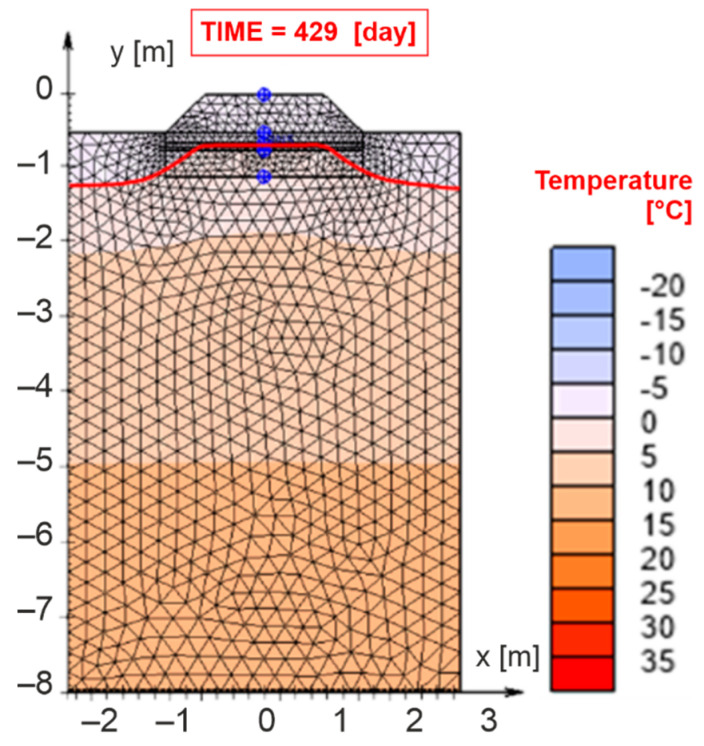
Results of numerical analysis.

In order to validate the numerical model, the thermal results of the experimental and numerical methods were compared. The values of the maximum air freezing index as well as the maximum freezing depth of the railway structure were reached on 5 March 2018, which in the numerical model represents the day TIME = 429 day. Figure 9 presents the maximum freezing depth of the structural layers *D_F,max_* = 0.690 m (marked by a red curve) obtained in the numerical model.

Based on the comparison of the maximum freezing depth of the railway line layers obtained by the experimental method (Figure 6b) and the numerical method (Figure 9), it can be observed that if real input parameters obtained in situ or in laboratory are used in the numerical modelling, it is possible to achieve a very good match of the monitored parameter. The difference between the values determined by the experimental and numerical methods is 0.002 m, which is a negligible difference with respect to the methodology of design of the structural sub-ballast layers of the railway line [100]. Moreover, the obtained accuracy of recreating the measurement results in the numerical model is the same as for other railway track substructure–subsoil systems [7] and consistent with previous observations [109]. Therefore, the model can be considered validated.

In the *SVHeat* program, in addition to monitoring the position of the zero-degree isotherm (freezing depth of the structure *D_F_*), it is also possible to determine the point temperature in individual material areas at different depths by defining a network of points of interest (see Figure 9—blue circles). Due to the possibility of comparing the real measured temperatures (measured in the experimental field) with the temperatures determined numerically, in the numerical model the points of interest were defined in places identical to the installation points of Pt1000 temperature sensors in the experimental field. The comparison of experimentally and numerically determined temperatures at the interface of individual material layers for the winter period 2017/2018 was presented in Table 5. The parameters *θ_i_* are temperatures determined by experimental method at the interface of individual material layers, *θ_2_* are temperatures at the sub-ballast upper surface, *θ_3_* are temperatures at the surface of the of the foamed concrete composite layer, *θ_4_* are temperatures at the lower edge of the composite layer of foamed concrete and *θ_5_* are temperatures at the level of the original subgrade surface. *θ_SVH,i_* represents temperatures determined by numerical method (subscript number characterizes the same level in construction as in the experimental method) and Δ*θ_i_* shows the difference between the temperatures obtained by the experimental and numerical methods. The differences between the temperatures determined by the experimental and numerical methods are in all points of interest (locations of temperature sensors) within the winter period 2017/2018 max. ±0.5 °C. The most important date is 5 March 2018 (TIME = 429 day), when the greatest freezing depth of the structural layers of the railway line was achieved. The performed comparison confirmed the correctness of the defined input parameters of numerical modelling, and therefore, they were used to create a numerical model burdened with more unfavorable environmental conditions.

The obtained temperature for the greatest freezing depth of the structural layers of the railway line achieved (5 March 2018; TIME = 429 day) was lower than for railway substructure solution with XPS layer (28 January 2019; TIME = 393 day) [7], but this is because in this study, the daily temperatures were lower (in this study, it was −1.1 °C, while Ižvolt et al. [7] assumed −0.4 °C). Another aspect is that, in this study, a high thermal conductivity coefficient was adopted for foam concrete compared to XPS [7], but it was caused by taking into account the actual ground conditions (moisture in the ground) and thus higher values of the thermal conductivity coefficient. The influence of humidity on the thermal conductivity coefficient of foam concrete is the subject of the present authors’ research. Moreover, the use of foamed concrete in railway substructure is aimed, in addition to improving heat transfer, also improving the bearing capacity of the subsoil, see Section 3.1.

### 3.3. Nomogram for the Design of the Modified Construction of Railway Sub-Ballast Layers

**Figure 10:**
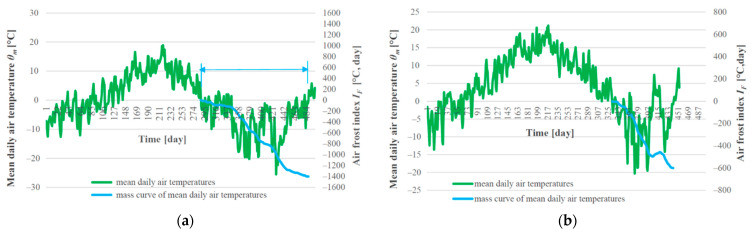
Graphical representation of selected climate characteristics used for numerical modelling air freezing index *I_F_* = 1400 °C, day and average annual air temperature *θ_m_* = 2 °C (**a**), *I_F_* = 600 °C, day and *θ_m_* = 6 °C (**b**).

The freezing subgrade surface in combination with unfavorable climate conditions (high value of air freezing index *I_F_*) result in the design of a large protective layer thickness *t_PL_* > 0.60 m, especially if it is realized from commonly used materials. Thus, it is appropriate to reduce the thickness of the protective layer by using thermal insulation materials, which not only save natural resources and reduce the thickness of the sub-ballast layers’ structure and they are inexpensive. Therefore, based on above numerical model, the numerical analysis of the impact of thermal insulation materials (composite layer of foamed concrete (FC) and extruded polystyrene (XPS)) on freezing of railway track was carried out, for cases of combination of unfavorable modelling input parameters (freezing subgrade, moderately polluted ballast bed, high values of air freezing index *I_F_*, and low values of average annual air temperatures *θ_m_*). The factors that most influence the freezing of the railway structure were analyzed in detail in the article [7]. Therefore, in this study, the impact of the most important factors influencing the freezing of the railway track (*I_F_* air freezing index, average annual air temperature *θ_m_*) on thermal behavior the railway track substructure–subsoil system was considered and a nomogram for designing necessary structural thicknesses of the modified sub-ballast layers with built-in thermal insulating materials (composite layer of FC, XPS) was created. In this purpose, the numerical analyses were carried out. Based on above, the model (2D) of a double-track railway line (as the most common case) situated on a 2.0 m high embankment was created. The most unfavorable climate conditions (air freezing index *I_F_* = 1400 °C, day and average annual air temperature *θ_m_* = 2 °C) were used in the numerical modelling. These values were determined from real measured values of mean daily air temperatures *θ_m_* obtained from various meteorological stations located in the Slovakia (see Figure 10).

The input material parameters of the numerical modelling are presented in Table 6. Opposite to the layers assumed in Section 2.2, XPS layer was added and instead of a clean ballast bed, a moderately polluted ballast bed was considered. Identical numerical analysis programs as described in Section 2.3 were used to perform the calculation of the freezing of the railway structure and to visualize the achieved results, with the same defined time step of the solution and boundary conditions of water freezing.

**Figure 11:**
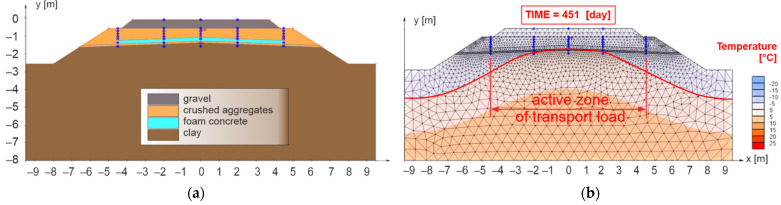
Numerical model No. 1—double-track railway with horizontal sub-ballast upper surface and with built-in composite layer of FC to the width of the active zone of traffic load for the applied climate conditions *I_F_* = 1400 °C, day and *θ_m_* = 2 °C (**a**), day of reaching the highest freezing depth of structural layers of numerical model (**b**).

In the first case (model No. 1, see Figure 11a), the sub-ballast layers, a 0.20 m thick composite layer of FC was designed for the width of the active zone of the traffic load (2.50 m from the axis of track) in combination with the layer of crushed aggregate fraction 0/31.5 mm. Moreover, the sub-ballast upper surface was designed as a horizontal and the subgrade surface with a roof slope of 5% (the most common case in the administration of the Slovak Railways [87]).

**Figure 12:**
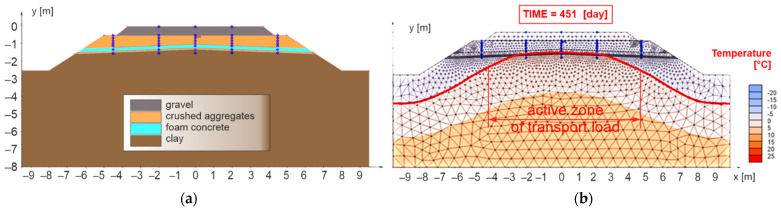
Numerical model No. 2—double-track railway line with horizontal sub-ballast upper surface and with built-in composite layer of FC extended to the whole width of the body of the railway bottom for the applied climate factors *I_F_* = 1400 °C, day and *θ_m_* = 2 °C (**a**), day of reaching the highest freezing depth of structural layers of the numerical model (**b**).

Figure 11b presents the results for the 451st day of numerical model No. 1, when the maximum freezing depth of the railway structure was reached (the zero-degree isotherm is highlighted by a red curve). It can be observed that the proposed thickness of the reinforcing and at the same time thermal insulation layer of FC is sufficient from the point of view of thermal insulation in the area of the axis of the subgrade of the double-track. In this case, however, there is a partial freezing of the subgrade surface in the area of the axis of the individual tracks and a significant freezing at the end of the active zone of the traffic load (approximately 2.50 m from the track axis). For these reasons, the numerical model No. 2 (Figure 12a) was created, where a composite layer of FC (reinforcing and at the same time thermal insulation layer) was placed on the levelling layer of the subgrade up to the face of the slope of the embankment.

**Figure 13:**
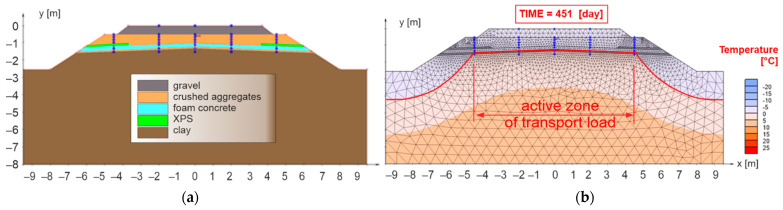
Numerical model No. 3—double-track railway with horizontal sub-ballast upper surface and with built-in composite layer of FC and XPS for applied climate conditions *I_F_* = 1400 °C, day and *θ_m_* = 2 °C (**a**), day of reaching the highest freezing depth of structural layers of the numerical model (**b**).

The extension of the composite layer of FC up to the face of the embankment improved the situation in the area of freezing in the axis of the earthwork (no freezing of the subgrade surface—Figure 12b), but there is still a significant freezing of the subgrade surface near the active traffic load zone (2.50 m from the track axis). Based on this, it was decided to incorporate another thermal insulation element (XPS) into the railway substructure—numerical model No. 3 (Figure 13a). In this case, the XPS was designed in a thickness of 0.10 m, and placed on the surface of the composite layer of FC up to a distance of 2.50 m (two plates of extruded polystyrene placed close together) from the face of the slope of the embankment.

**Figure 14:**
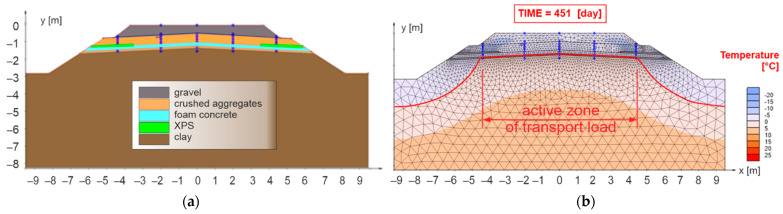
Numerical model No. 4—double-track railway with inclined sub-ballast upper surface and with built-in composite layer of FC and XPS for applied climate conditions *I_F_* = 1400 °C, day and *θ_m_* = 2 °C (**a**), day of reaching the highest freezing depth of structural layers of the numerical model (**b**).

Figure 13b demonstrates appropriate composition of the sub-ballast layers of the model No. 3, since the freezing of the subgrade surface did not occur within the entire profile of the active zone of traffic load. As it is a common practice abroad to use a roof-like slope also for the sub-ballast upper surface, and also due to greater savings of natural material (crushed aggregates, gravels), another numerical model was created, designated as No. 4 (Figure 14a). From this picture is visible that, as in the previous case (model No. 3), the subgrade surface did not freeze within the entire profile of the active traffic load zone (Figure 14b). Therefore, numerical model No. 4 was used to compile a design nomogram for the selection of necessary construction thicknesses of the modified structure of sub-ballast layers with built-in thermal insulation materials—composite layer of FC and XPS.

A total of 40 numerical models of the railway track substructure–subsoil system were created. The individual numerical models differed from each other by different construction thickness of the protective and thermal insulation layer (composite layer of FC and XPS layers), the thickness of which changed due to different climate conditions (air freezing index and average annual air temperature). The designed thickness of the composite layer of FC was reduced with decreasing intensity of climate parameters (*I_F_* < 1400 °C, day, *θ_m_* > 2 °C), and design thicknesses of 0.10 m, 0.15 m, and 0.20 m were considered as final. In the same way, the required construction thickness of the XPS layer was changed, with final construction thicknesses of 0.05 m, 0.06, 0.08 m, and 0.10 m. The thickness of the protective layer of crushed aggregate, composite layer of FC, and plates of XPS (these three structural layers as a whole form the protection of the subgrade surface against freezing) in individual numerical models was adjusted so that on the day of reaching the greatest depth of freezing of the railway structure, the freezing of the subgrade surface in the active zone of the traffic load would not occur. Freezing of the subgrade surface in the area of the active zone of traffic load could cause in winter an uneven lift of the railway track and in spring time decrease of its bearing capacity with adverse effects on the required geometric position of the track according to [100].

**Figure 15:**
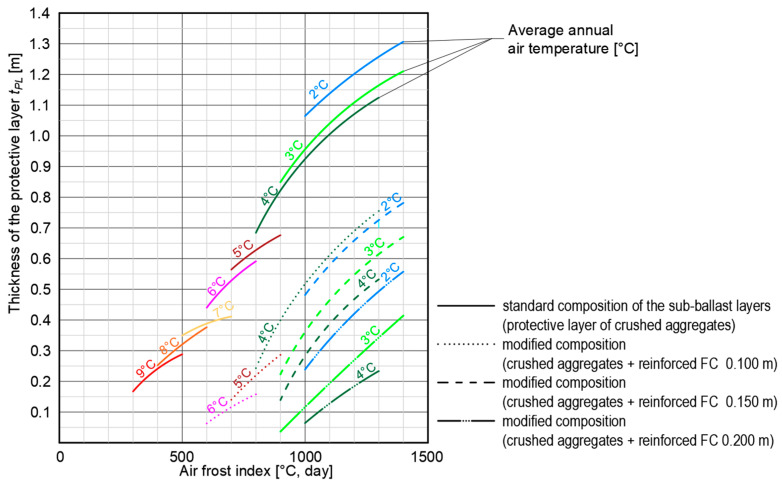
Design nomogram—dependence of the thickness of the protective layer made of crushed aggregate on the climate conditions for the railway sub-ballast layers with the built-in thermal insulation layer made of composite FC.

Based on the results of numerical analyses, a nomogram of the dependence of the construction thickness of the protective layer of the freezing subgrade surface (crushed aggregate layer combined with thermal insulation materials—FC and XPS plates) on climate conditions (*I_F_*, *θ_m_*) was compiled and is depicted in Figure 15.

Due to the complexity of using the nomogram for design of the structural sub-ballast layers with regards to the non-traffic load (climate factors) and the possibility of assessing the reduction of the crushed aggregate layer, the nomogram also adds the dependence of the construction protective layer thickness of the sub-ballast layers without thermal insulation layer.

Based on the value of the air freezing index (horizontal axis on Figure 15) of the area of interest and average annual air temperature (skew lines), the required thickness of the protective layer (*t_PL_*) and thermal insulation layer of composite FC (*t_FC_*) can be assessed. Horizontal lines characterize the required construction thickness of the protective layer of crushed aggregate (*t_PL_*) and different types of lines characterize the required construction thickness of the thermal insulation layer of composite FC (solid line—without thermal insulation layer).

It should be noted that in order to protect the subgrade surface against freezing from the slope of the embankment body (in the area of the active zone of traffic load), it is necessary to design extra plates of XPS in the construction of the sub-ballast layers. The thickness and location of the XPS boards (placed directly on the surface of the composite FC layer) must be designed with respect to the climate conditions, in the following design thicknesses, which are based on the assumed air freezing index:

- if *I_F_* ≤ 800 °C, day, *θ_m_* ≥ 5 °C, XPS plates of thickness 0.05 m are required up to 1.85 m from the edge of the embankment slope;
- if *I_F_* ≤ 1000 °C, day, *θ_m_* ≥ 3 °C, XPS plates of thickness 0.06 m are required up to 2.50 m from the edge of the embankment slope;
- if *I_F_* ≤ 1200 °C, day, *θ_m_* ≥ 2 °C, XPS plates of thickness 0.08 m are required up to 2.50 m from the edge of the embankment slope;
- if *I_F_* ≤ 1400 °C, day, *θ_m_* ≥ 2 °C, XPS plates of thickness 0.10 m are required up to 2.50 m from the edge of the embankment slope.

At the same time, it should be emphasized that in order to protect the surface of composite FC layer from dynamic impact from rail transport, it is recommended to design a thickness of crushed aggregate protective layer of at least 0.25 m (min. 0.15 m on the surface of composite FC and 0.10 m below FC layer as a levelling layer).

### 3.4. Mathematical Model for the Design of the Modified Structure of the Sub-Ballast Layers

The obtained data from the experimental measurements (Figure 3, Figure 5 and Figure 10) and numerical analyses (Section 3) of the railway line for climate conditions (non-traffic load) can be used to create a mathematical model for the possibility of continuous monitoring of changes in the freezing of the railway structure depending on climate characteristics such as air freezing index and average annual air temperature.

Based on the distribution of experimentally and numerically obtained data and the discrete dependence of the freezing depth of the railway line *D_F_* on the value of the air freezing index *I_F_* and the average annual air temperature *θ_m_*, the formula for determining *D_F_* can be approximated by a power function:

(2) $$D_{F}=c\theta_{m}^{a}I_{F}^{b},\;\;\mathrm{where}\;\theta_{m}>0,\;I_{F}>0.$$

To determine the unknown coefficients *a*, *b*, *c*, a sufficiently stable least squares method shall be used, where the sum of the squares of the deviations between the obtained and approximated data is to be minimal, and thus the minimum of the sum function is sought:

(3) $$S\left(a,b,c\right)=\sum_{i=1}^{n}{\left(c\theta_{mi}^{a}I_{Fi}^{b}-D_{Fi}\right)}^{2}.$$

After linearizing of this problem and denoting $C=\ln\;c,x_{i}=\ln\theta_{mi},\;y_{i}=\ln I_{Fi\;},\;f_{i}=\ln D_{Fi}\;,\;i=1,\;\ldots n$ we obtain a set of linear equations:

(4) $$\begin{array}{c} \mathrm{where}C=(\sum_{i=1}^{n}f_{i}-a\sum_{i=1}^{n}x_{i}-b\sum_{i=1}^{n}y_{i})/n\;,aQ_{xy}+bR_{y}-Q_{fy}=0,aR_{x}+bQ_{xy}-Q_{fx}=0,\\ \mathrm{where}\;Q_{uv}=(n\sum_{i=1}^{n}u_{i}v_{i}-\sum_{i=1}^{n}u_{i}\sum_{i=1}^{n}v_{i})\;,\;R_{u}=n\sum_{i=1}^{n}u_{i}^{2}-{\left(\sum_{i=1}^{n}u_{i}\right)}^{2}.\; \end{array}$$

Solving the Formula (4) yields the following coefficients:

(5) $$\;c=e^{C}.$$

The global of this approximation is defined from the following equation:

(6) $$\varepsilon =\sqrt{\overset{n}{\underset{i=1}{\sum }}{\left(c\theta_{mi}^{a}I_{Fi}^{b}-D_{Fi}\right)}^{2}/n}$$

Depending on the value of the air freezing index and the average annual air temperature that were entered into the numerical model and which also affect the freezing depth of the railway structure, the approximation function sought for continuous monitoring of the railway line freezing depth is generally expressed in the Equation (7).

(7) $$D_{F}=c_{1}\theta_{m}^{a_{1}}I_{F}^{b_{1}}+c_{2}I_{F}^{2}+c_{3}I_{F}+c_{4},$$

where coefficients *a*_1_, *b*_1_, *c*_1_ are specifically obtained using the approach mentioned above. Coefficients *c_j_* for *j* = 2, 3, 4 can also be determined using the least squares method, where for numerical data $h_{i\;},$ a minimum of a particular sum function is sought after:

(8) $$S\left(c_{2},c_{3},c_{4}\right)=\overset{n}{\underset{i=1}{\sum }}{\left(c_{2}I_{Fi}^{2}+c_{3}I_{Fi}+c_{4}-h_{i}\right)}^{2}.$$

The solution of this are coefficients in the following form (using the denotation from Equation (5)):

(9) $$c_{2}=(Q_{xh}Q_{x^{2}x}-R_{x}Q_{hx^{2}})/\left(Q_{xx^{2}}^{2}-R_{x}R_{x^{2}}\right),\;c_{3}=(Q_{x^{2}x}Q_{x^{2}h}-R_{x^{2}}Q_{hx})/\left(Q_{xx^{2}}^{2}-R_{x}R_{x^{2}}\right),\;c_{4}=(\sum_{i=1}^{n}h_{i}-c_{2}\sum_{i=1}^{n}x_{i}^{2}-c_{3}\sum_{i=1}^{n}x_{i})/n\;$$

In the case of specific environmental conditions (*I_F_* ≥900 °C, day a *θ_m_* < 5 °C) and reduction of the protective layer of crushed aggregate using a thermal insulation composite foamed concrete layer of thickness *z_i_* = 200 mm *c_j_* = *0* for *j* = 2, 3, 4 in Equation (7) and for calculating the freezing depth of the railway structure or required thickness of the protective layer, the following relations are used:

(10) $$D_{F}=c_{1}\theta_{m}^{a_{1}}I_{F}^{b_{1}}=0.0025\theta_{m}^{-0.32}I_{F}^{0.891}$$

(11) $$t_{PL}\;=D_{F}-0.7\;=\;0.0025\theta_{m}^{-0.32}I_{F}^{0.891}-0.7.\;$$

Global error for this specific mathematical model is $\varepsilon \dot{=}0.0117$.

In the case of environmental conditions *I_F_* ≤ 900 °C, day and *θ_m_* ≥ 3 °C and construction of the protective layer of the subgrade surface only from crushed aggregate (without the use of a thermal insulation layer in the sub-ballast layers), the following relationships apply for calculating the freezing depth of the railway structure or the required thickness of the protective layer:

(12) $$D_{F}=0.1436\theta_{m}^{-0.28}I_{F}^{0.374},\;$$

(13) $$t_{PL}\;=D_{F}-0.5=0.1436\theta_{m}^{-0.28}I_{F}^{0.374}-0.5.$$

With regard to all assessed parameters of the railway line construction is the mathematical model for *I_F_* > 900 °C, day and *θ_m_* < 5 °C expressed as:

(14) $$D_{F}=0.2808\theta_{m}^{-0.33}I_{F}^{0.3},$$

(15) $$t_{PL}\;=D_{F}-0.5=0.2808\theta_{m}^{-0.33}I_{F}^{0.3}-0.5.\;$$

It is important to note that in the procedure of calculating the freezing depth of the railway line *D_F_* or the required thickness of the crushed aggregate protective layer *t_PL_* the boundary conditions (*I_F_* and *θ_m_*) of the calculation must be taken into account as $I_{F}\;=$ 900 °C, day causes a change in the relations for their calculation. Particularly the air freezing index $I_{F}\;=$ 900 °C, day is characterized in the numerical method by a change in the freezing period (significant decrease of days warm days with a temperature above 0 °C within the freezing period—numerical model used real data obtained from meteorological stations), which was also found in the mathematical models. The global error for both of these mathematical models is $\varepsilon \dot{=}0.0117$. Table 7 displays the difference Δ*D_F_* = *D_F,mat_* − *D_F,num_* between the calculated data using the mathematical model (Equations (10), (12) and (14)) and data obtained using numerical modelling found in Section 3.

In the case of sub-ballast layers with built-in thermal insulation layer composed of composite FC of thickness *z_i_* = 200 mm are the deviations between the data determined with numerical and mathematical method in the interval 〈−0.011;0.017〉. In the case that no thermal insulation layer of composite FC used, the deviations for *I_F_* ≤ 900 °C, day are in the interval 〈−0.022;0.018〉 and for *I_F_* > 900 °C, day are in the interval 〈−0.024;0.018〉.

The values determined using a mathematical model were rounded to two decimal places. Subsequently, when rounding up to a number divisible by five, the same values were obtained as when the numerically obtained data were rounded up also to a multiple of 50 mm. The mathematical model thus describes with sufficient accuracy and continuously the depth of freezing of the railway track as well as the required thickness of the protective layer of the railway track structure.

Different mathematical models are used for the reduction of the protective layer made up of crushed aggregate and composite FC of thickness *z_i_* = 100 mm depending on the air freezing index *I_F_*. For *I_F_* > 900 °C, day, it is assumed that based on experimental data and real conditions $\theta_{m}=4\;{}^{\circ}C.$ In the Equation (7) $c_{1}=0$ and other coefficients are determined using the relations in Equation (9).

To calculate the freezing depth of the railway line structure or the required thickness of the protective layer, the following relations are used:

(16) $$D_{F}=10^{-6}\left(0.725I_{F}^{2}+2424.5I_{F}\right)-0.56855,$$

(17) $$t_{PL}=D_{F}-0.60=10^{-6}\left(0.725I_{F}^{2}+2424.5I_{F}\right)-1.16855.$$

The global error for this specific mathematical model is $\varepsilon \dot{=}0.007$. Deviations Δ*D_F_* between data obtained numerically and mathematically are in the interval 〈−0.01;0.01〉.

For *I_F_* ≤ 900 °C, day and $\theta_{m}\geq \;4\;{}^{\circ}C$, in Equation (7)$\;c_{j}=0$ for j=2,3,4 and the functions determining the dependence of freezing depth of railway line structure and the necessary thickness of the protective layer are in the form:

(18) $$D_{F}=0.0393\theta_{m}^{-0.43}I_{F}^{0.555},$$

(19) $$t_{PL}\;=\;D_{F}-0.6=0.0393\theta_{m}^{-0.43}I_{F}^{0.555}-0.6.$$

The deviations Δ*D_F_* between data obtained from numerical and mathematical models are in the interval 〈−0.026;0.03〉 and global error for the mathematical model is $\varepsilon \dot{=}0.024$.

In case of unfavorable environmental conditions (*I_F_* > 900 °C, day and *θ_m_* < 5 °C) and reduction of a part of the protective layer from crushed aggregate by means of thermal insulation layer made up composite foamed concrete with thickness *z_i_* = 150 mm are based on Equation (7) coefficients $\;c_{j}=0$ for j=2,3,4. According to the above-mentioned procedure for the calculation of the freezing depth of the railway track structure or the required thickness of the protective layer, the approximation functions are expressed as follows:

(20) $$D_{F}=0.006667\theta_{m}^{-0.24}I_{F}^{0.767},\;$$

(21) $$t_{PL}=D_{F}-0.65=0.006667\theta_{m}^{-0.24}I_{F}^{0.767}-0.65.\;$$

Global errors for approximation functions defined in this manner is $\varepsilon \dot{=}0.014$. Deviations Δ*D_F_* between the data obtained mathematically and numerically are in the interval 〈−0.0196; 0.019〉.

The approximation functions for calculating the freezing depth of the railway structure depending on the average annual air temperature and the air freezing index are sufficient, as the thickness of the protective layer of crushed aggregate is still rounded up to a full 50 mm. The size of the global error of the calculation is approximately 20 mm and this ensures that after rounding the thickness of the protective layer from the crushed aggregate, the same final values are obtained as after rounding the values from experimental measurements and numerical modelling.

## 4. Conclusions

The aim of the article was the optimization of railway track substructure by using innovative layers system to reduce the consumption of natural aggregate as an exhausting resource and the costs. Composite foamed concrete with XPS plates was used in different structure compositions in the experimental testing field at scale 1:1. In order to optimize the structure for different climate conditions, four FEM numerical models were created and studied. Based on the obtained results of experimental and mathematical and numerical analyses, it can be stated that:

1. Application of composite FC in the railway sub-ballast layers makes it possible to increase its bearing capacity (see Figure 8). In case of a subgrade with sufficiently high bearing capacity (static modulus of deformation *E_0_* = 40 ± 2 MPa), it is possible through application of a composite FC layer of thickness 0.10 m to increase the value of static modulus of deformation after 28 days of its maturation twice (*E_mat_* > 80 MPa). After two winter periods, it is possible to detect up to 3.5 times the value of the original static modulus of deformation (*E_mat_* > 140 MPa) on the surface of composite foamed concrete. In the case of a subgrade with low bearing capacity (static modulus of deformation *E_0_* = 10 ± 2 MPa), it is possible through application of a composite FC layer of thickness 0.15 m to increase the value of static modulus of deformation after 28 days of its maturation by ten-times (*E_mat_* > 100 MPa). After two winter periods, it is possible to detect up to 18 times the value of the original static modulus of deformation (*E_mat_* > 180 MPa) on the surface of composite FC. The use of composite FC therefore seems to be suitable especially in the case of subgrades with low bearing capacity, where a reduction in the thickness of the sub-ballast (saving natural materials—gravels or gravel-sand) and a long-term guarantee of the required geometric position of the track is expected according to [100].
2. The system of the modified construction of the sub-ballast layers for the double-track railway (application of composite foamed concrete—see Figure 8 and Figure 9) allows a significant reduction of the protective layer consisting of natural materials (crushed aggregates or gravel-sand), especially in areas with unfavorable climate conditions (*I_F_* > 1000 °C, day and *θ_m_* < 4 °C). For example, for air freezing index *I_F_* = 1000 °C, day and average annual air temperature *θ_m_* = 3 °C it is possible to save a thickness of 0.60 m of natural materials and replace the crushed aggregate or gravel-sand layer with a layer of composite foamed concrete of thickness 0.15 m. In case of air freezing index *I_F_* = 1400 °C, day and average annual air temperature *θ_m_* = 2 °C, it is possible to save a thickness of 0.75 m of natural resources and replace these with a layer of composite foamed concrete with a thickness of 0.20 m (see Figure 15).
3. In the case of a combination of freezing susceptible soils in the subgrade surface of the railway line and unfavorable environmental conditions (*I_F_* > 600 °C, day and *θ_m_* < 6 °C), there is a significant penetration of frost to the subgrade surface from the track bench and the slope of the embankment. In order to protect the subgrade surface from freezing, it is necessary to ensure that the zero-degree isotherm does not fall below the subgrade surface level in the entire active zone of the traffic load. For example, as depicted in Figure 12b, it is possible to observe that in the area of the track axis there was no freezing of the subgrade surface, but at the end of the active zone (in this case it is about 2.50 m from the track axis), significant freezing can be seen. For this reason, in addition to composite FC, it is appropriate to apply XPS plates (Figure 13) in the structural composition of the railway sub-ballast layers, which will prevent this unfavorable phenomenon.
4. Due to greater savings of natural resources (gravels or crushed aggregates), a different way of implementing the design of the sub-ballast upper surface (horizontal—Figure 10 or in a roof-like 5% slope—Figure 14) was assessed within the numerical modelling. From the point of view of the protection of the subgrade surface against freezing in the area of the active zone of the traffic load, both of these cases of implementation can be considered practically equivalent. Figure 14 (numerical model No. 4 with a roof-like way of implementing the sub-ballast upper surface design) was therefore chosen as a reference (allows greater savings of sub-ballast materials and at the same time increasing the volume of material behind the sleepers leads to better contactless track stability).
5. The nomogram shown in Figure 15 was created to determine the required construction thickness of composite FC and the protective layer of crushed aggregates depending on the climate conditions (non-traffic load). In addition, to protect the subgrade surface against freezing in the entire active zone, it is also necessary to design different thicknesses and locations of the XPS plates. The detailed design procedure is described in Section 3.
6. The relations given in Section 4 (Equations (10)–(21)) were determined to estimate the freezing depth of the railway structure or the required thickness of the protective layer of crushed aggregate for the standard structure of the sub-ballast layers (without the use of thermal insulation material in the sub-ballast layers) or for the modified structure of the sub-ballast layers (installation of 0.10 m, 0.15 m, or 0.20 m thick composite FC layers). Within the design, it is necessary to take into account the boundary conditions of the calculation, as during the course of winter period at the value of the air freezing index *I_F_* = 900 °C, day, there is a change in the relations for calculating the necessary parameters.

As part of further experimental activities of Department of Railway Engineering and Track Management of University of Žilina, the presented modified composition of the body of the railway sub-ballast layers will be installed in real operating conditions in the network of Slovak Railways. The aim is to verify the results presented in this article (experimental and mathematical analysis and numerical modelling), and thus to confirm the positive impact of composite FC layers built into the railway line sub-ballast layers, which is constantly affected by increased traffic and non-traffic load with low bearing capacity of the subgrade. The positive results achieved even in such operational, geotechnical, and environmental conditions will significantly affect not only the saving of natural finds of building materials, but will also increase the stability and service life of the railway line to which composite foamed concrete layers will be applied. In addition, as part of future research, it is planned to research similar solutions in other infrastructures, such as road pavements or slabs on the ground and to determine the similar—as in this study—monogram to define the thickness of the protective layer made of crushed aggregate dependence on the climate conditions for the subbase layers with the built-in thermal insulation layer made of composite FC.

## Figures and Tables

**Table 1:** Strength and physical parameters of used materials.

Material	Density (kg/m^3^)	Elasticity Modulus (MPa)	Poisson’s Ratio (−)	Compressive Strength (MPa)	Flexural Strength (kN/m)	Tensile Strength (kN/m)
Gravel fr. 31.5/63 mm	1900	-	0.15 ^1^	-	-	-
Crushed aggregate fr. 0/31.5 mm	1930	-	0.20 ^1^	-	-	-
Composite FC 600	600	1400	0.22	2.0	0.5	-
Geofiltex 63/20 T	200 ± 20 ^2^	-	-	-	-	5.0 ^3^

^1^ values assumed in numerical model based on soil class and consistency and experience of authors. ^2^ area weight 200 ± 20 g/m^2^ according to EN ISO 9864; source [95]. ^3^ tensile strength of geotextile was inserted from certificate of producer according to EN ISO 10319; source: [95].

**Table 2:** Properties of material layers and subsoil.

Construction Layer/Material Characteristics	Ballast Bed—New	Protective Layer	Reinforcing, Thermal Insulation Layer	Levelling Layer	Subsoil
Material of the layer	gravel fr. 31.5/63 mm	crushed aggregate fr. 0/31.5 mm	composite FC 600	crushed aggregate fr. 0/31.5 mm	clay
Bulk density (kg/m^3^)	1900	1930	600	1930	1650
Specific heat capacity (J/(kg·K))	980	1090	1100	1090	1095
Thermal conductivity coefficient (W/(m·K))	0.7	1.73	0.25	1.73	1.55

**Table 3:** Climate parameters of individual winter periods of segment A at the experimental field.

Winter Period	*θ_s,max_* (°C)	*θ_s,min_* (°C)	*θ_m_* (°C)	*I_F_* (°C, day)	*I_Fs_* (°C, day)	*D_F_* (m)
2017/2018	8.1	−11.2	9.0	107	98	0.692

**Table 4:** Input parameters of numerical modelling—results of measurement.

Construction Layer/Material Characteristics	Ballast Bed—New	Protective Layer	Reinforcing, Thermal Insulation Layer	Levelling Layer	Subsoil
Material of the layer	gravel fr. 31.5/63 mm	crushed aggregate fr. 0/31.5 mm	composite FC 600	crushed aggregate fr. 0/31.5 mm	clay
Temperature (°C)	−2	3	4	5	10
Humidity (%) ^1^	1	5.5	30	5.5	26

^1^ Humidity was measured by TDR sensors in certain depth of the model.

**Table 5:** Comparison of temperatures at the interface of individual sub-ballast layers determined by experimental and numerical method.

Date (Time in Numerical Model)	*θ*_2_ (°C)	*θ_SVH,2_* (°C)	∆*θ_2_* (°C)	*θ_3_* (°C)	*θ_SVH,3_* (°C)	∆*θ_3_* (°C)	*θ_4_* (°C)	*θ_SVH,4_* (°C)	∆*θ_4_* (°C)	*θ*_5_ (°C)	*θ_SVH,5_* (°C)	∆*θ_5_* (°C)
4 Febuary 2018 (400)	2.79	3.00	0.21	3.02	3.10	0.08	4.09	4.00	−0.09	4.72	4.40	−0.32
9 Febuary 2018 (405)	1.76	1.90	0.14	2.04	2.00	−0.04	3.56	3.40	−0.16	4.38	3.90	−0.48
14 Febuary 2018 (410)	1.82	2.30	0.48	2.08	2.40	0.32	3.39	3.40	0.01	4.12	3.80	−0.32
19 Febuary 2018 (415)	1.62	2.10	0.48	1.90	2.30	0.40	3.24	3.30	0.06	3.94	3.70	−0.24
24 Febuary 2018 (420)	1.23	1.60	0.37	1.36	1.70	0.34	2.88	3.00	0.12	3.64	3.50	−0.14
1 March 2018 (425)	−0.56	−0.7	−0.14	−0.27	−0.7	−0.43	1.94	1.70	−0.24	2.99	2.60	−0.39
5 March 2018 (429)	−1.12	−0.90	0.22	−0.78	−0.80	−0.02	1.20	1.20	0.00	2.34	1.90	−0.44
6 March 2018 (430)	−0.86	−0.80	0.06	−0.54	−0.60	−0.06	1.20	1.20	0.00	2.26	1.90	−0.36
11 March 2018 (435)	0.06	0.00	−0.06	0.05	0.20	0.15	1.52	1.50	−0.02	2.29	2.00	−0.29
16 March 2018 (440)	3.59	3.80	0.21	3.38	3.70	0.32	3.23	3.20	−0.03	3.18	3.00	−0.18
21 March 2018 (445)	0.99	1.10	0.11	1.31	1.20	−0.11	2.70	2.60	−0.10	3.35	3.00	−0.35
26 March 2018 (450)	1.63	1.50	−0.13	1.74	1.50	−0.24	2.54	2.30	−0.24	3.08	2.70	−0.38

**Table 6:** Input parameters of numerical modelling—material characteristics.

Construction Layer/Material Characteristics	Ballast Bed—Moderate Pollution	Protective Layer	Reinforcing, Thermal Insulation Layer	Thermal Insulation Layer	Levelling Layer	Subsoil
Material of the layer	gravel fr. 31.5/63 mm	crushed aggregate fr. 0/31.5 mm	composite foamed concrete FC 600	extruded polystyrene	crushed aggregate fr. 0/31.5 mm	clay
Temperature (°C)	−2	3	4	−2	5	10
Humidity (%)	4	5.5	30	12	5.5	26
Bulk density (kg·m^−3^)	1900	1930	600	35	1930	1650
Specific heat capacity (J·kg^−1^·K^−1^)	980	1090	1100	2060	1090	1095
Thermal conductivity coefficient (W·m^−1^·K^−1^)	1.0	1.73	0.25	0.04	1.73	1.55

**Table 7:** Deviations of the values determined by the mathematical and numerical method for the freezing depth of railway line construction *D_F_* depending on air freezing index *I_F_* and average annual air temperature *θ_m_* (sub-ballast layers with a built-in thermal insulation layer made of composite foamed concrete 200 mm thick and also without thermal insulation layer).

Without Thermal Insulation Layer *z_i_* = 0 mm					With Thermal Insulation Layer *z_i_* = 200 mm				
*I_F_* (°C, Day)	*θ_m_* (°C)	*D_F,mat._* (m)	*D_F,num._* (m)	Δ*D_F_* (m)	*I_F_* (°C, day)	*θ_m_* (°C)	*D_F,mat._* (m)	*D_F,num._* (m)	Δ*D_F_* (m)
1000	2.0	1.560	1.564	0.002	1000	2.0	0.940	0.933	0.007
1100	2.0	1.620	1.642	−0.022	1100	2.0	1.030	1.027	0.003
1200	2.0	1.690	1.196	−0.006	1200	2.0	1.110	1.120	−0.010
900	3.0	1.340	1.338	0.002	1300	2.0	1.190	1.175	0.015
1000	3.0	1.480	1.470	0.010	1400	2.0	1.270	1.264	0.006
1100	3.0	1.550	1.542	0.008	900	3.0	0.750	0.738	0.012
1000	4.0	1.430	1.428	−0.015	1000	3.0	0.830	0.817	0.013
700	5.0	1.060	1.064	−0.004	1100	3.0	0.900	0.888	0.012
800	5.0	1.110	1.129	−0.019	1200	3.0	0.970	0.981	−0.011
900	5.0	1.170	1.176	−0.006	1300	3.0	1.050	1.034	0.016
600	6.0	0.950	0.944	0.006	1400	3.0	1.120	1.113	0.007
700	6.0	1.010	1.026	−0.016	1000	4.0	0.760	0.768	−0.008
500	7.0	0.850	0.849	0.001	1100	4.0	0.820	0.819	0.001
400	8.0	0.750	0.752	−0.002	1200	4.0	0.890	0.883	0.007
500	8.0	0.820	0.820	0.000	1300	4.0	0.950	0.933	0.017

## Data Availability

The data presented in this study are available on request from the corresponding author. At the time the project was carried out, there was no obligation to make the data publicly available.
